# Sex and age specific reduction in stress resistance and mitochondrial DNA copy number in *Drosophila melanogaster*

**DOI:** 10.1038/s41598-019-48752-7

**Published:** 2019-08-23

**Authors:** Torsten Nygaard Kristensen, Volker Loeschcke, Qihua Tan, Cino Pertoldi, Jonas Mengel-From

**Affiliations:** 1 Section of Biology and Environmental Science, Department of Chemistry and Bioscience, Aalborg University, Frederik Bajers Vej 7H, Aalborg, DK 9220 Denmark; 2 Department of Bioscience, Aarhus University, Ny Munkegade 116, DK-8000 Aarhus C, Denmark; 3 The Danish Aging Research Center and The Danish Twin Registry, Epidemiology Unit, Institute of Public Health, University of Southern Denmark, J.B. Winsløws Vej 9, DK-5000 Odense, Denmark; 4 Department of Clinical Genetics, Odense University Hospital, Sdr. Boulevard 29, DK-5000 Odense, Denmark; 5 Aalborg Zoo, Mølleparkvej 63, DK-9000 Aalborg, Denmark; 6 Clinical Biochemistry and Pharmacology Odense University Hospital, J. B. Winsløws Vej 4, DK-5000 Odense C, Denmark

**Keywords:** Evolution, Physiology

## Abstract

Environmental stresses such as extreme temperatures, dehydration and food deprivation may have distinct consequences for different age-classes and for males and females across species. Here we investigate a natural population of the model organism *Drosophila melanogaster*. Males and females at ages 3, 19 and 35 days were tested for stress resistance; i.e. the ability of flies to cope with starvation and both cold and hot temperatures. Further, we tested a measure of metabolic efficiency, namely mitochondrial DNA copy number (mtDNA CN) in both sexes at all three age-classes. We hypothesize that stress resistance is reduced at old age and more so in males, and that mtDNA CN is a biomarker for sex- and age-dependent reductions in the ability to cope with harsh environments. We show that: (1) males exhibit reduced starvation tolerance at old age, whereas older females are better in coping with periods without food compared to younger females, (2) heat tolerance decreases with increasing age in males but not in females, (3) cold tolerance is reduced at old age in both sexes, and (4) old males have reduced mtDNA CN whereas mtDNA CN slightly increases with age in females. In conclusion, our data provide strong evidence for trait and sex specific consequences of aging with females generally being better at coping with environmental stress at old age. The reduced mtDNA CN in old males suggests reduced metabolic efficiency and this may partly explain why males are less stress tolerant at old age than females. We suggest that mtDNA CN might be a suitable biomarker for physiological robustness. Our findings likely extend to other taxa than *Drosophila* and therefore we discuss the observations in relation to aging and sex specific lifespan across species.

## Introduction

Abiotic environmental factors such as extreme temperature and starvation are known to cause stress to organisms and reduce their physiological performance. Environmental stress can be defined as factors that lead to marked decreases in fitness^1^. It has been shown in numerous studies on model species, such as *Caenorhabditis elegans* and *Drosophila melanogaster*, that the ability to cope with some abiotic stressful conditions is reduced at old age^2–7^. This phenomenon has been linked to functional senescence describing malfunctions that progressively occur near the end of life^3,8^. In this paper aging is defined according to Rose^9^ as “a persistent decline in the age-specific fitness components of an organism due to internal physiological degeneration.”

Aspects of environmental stress research that are less explored include the interactions between harsh environmental conditions, sex and age, and the mechanisms explaining possible sex and age-specific abilities to cope with environmental stress. Sex specific lifespan is a common phenomenon across many species^10,11^. Evolutionary hypotheses accounting for sex specific lifespan and aging include differential vulnerability to environmental stress, differential intensity of sexual selection, partly distinct genetic architectures, and distinct patterns of parental care and investment in reproduction^11,12^. Empirical studies testing these ideas often focus on hormones, asymmetric inheritance of sex chromosomes and sex-specific regulation of the mitochondria^13,14^. The hypotheses that can explain differential lifespan in males and females may also explain differences in stress tolerance between sexes at old age. First, lower lifespan and robustness at old age in the heterogametic sex, may be partly explained by the fact that recessive deleterious mutations on the X (or Z) chromosomes will typically be expressed only in the heterogametic sex^10,14^. This is because such mutations are often rare, thus the likelihood of homozygosity for genes on the sex chromosomes in the homogametic sex is low. Thus this hypothesis can explain lower lifespan in males (or more generally the heterogametic sex) compared to females, and also why males may suffer more from environmental stress at old age compared to females if the maladaptive influence of these alleles is more pronounced at old age. Second, maternal inheritance of mitochondrial DNA, can cause accumulation of deleterious mutations in the mitochondrial genome of males, leaving females with better control over mitochondrial functions and maintenance^14^. This may also result in increased male mortality and potentially lower the ability to cope with environmental stress at old age in males compared to females^10,14,15^. However, these hypotheses have received relatively little empirical support and further studies are needed to understand the basis of sex differences in aging and longevity, which could reveal novel mechanisms underlying intraspecific variability in aging rate^11,16^. Further knowledge on sex- and age-specific stress resistance is important, when trying to understand the impact of environmental change on different age-classes and sexes in natural populations. This may be especially important for long-lived species where evolutionary speed is typically slower due to longer generation times. A change in age-class and sex distributions of natural populations in increasingly stressful environments is expected to impact on e.g. reproductive output, the speed of evolutionary processes and effective population sizes, and thereby also on the likelihood of adapting to, and thriving in, rapidly changing environments.

Aging and lifespan are quantitative traits influenced by the combined effect of many genes and the environment. Estimates of heritabilities of lifespan obtained from studies on model species are typically in the range 0.2–0.4^17^. Lehtovaara *et al*.^12^ studied the genetic architecture of lifespan and aging in *D*. *melanogaster* and they showed that a large proportion (three-quarters) of the additive genetic variation for these traits is sex specific. This has several important evolutionary implications and suggests that the traits can evolve partly independently in males and females. Genomic studies, including GWAS, have revealed mechanisms and candidate genes explaining variation in lifespan in humans^18^. These include genes involved in lipid and carbohydrate metabolism^19^, cardiovascular genes such as *APOE*, which has an important role in regulating lipoproteins^20^, immune system genes^21^, and telomere length^22^. Also, mitochondrial mutations and mitochondria DNA copy numbers have been proposed to be important for lifespan^23–25^. Despite numerous studies within this research field, they typically do not focus on sex specific genetic architectures of aging and lifespan^26^.

Mitochondria DNA copy number (mtDNA CN) has been proposed to constitute an age-related biomarker of general health in humans^23,24^. mtDNA CN is a relative measure of the content of mtDNA relative to nuclear DNA. This proxy measures the amount of mitochondria in cells, although the mtDNA content may vary between cells, especially in different tissues. mtDNA CN has likewise been shown to be influenced by environmental factors such as oxidative stress, diet composition and toxins across multiple species^27,28^. In *D*. *melanogaster* age and sex also influence the mitochondria and mtDNA CN^28–31^.

Here we investigate the impact of age and sex on tolerance to acute exposure to heat, cold and starvation stress in male and female *D*. *melanogaster* being 3, 19 or 35 days old. Further, we investigate mtDNA CN in both sexes and all three age-classes with the aim of deducing how age and sex-specific stress tolerance associate with mtDNA CN. Doing this we test the potential of mtDNA CN as a biomarker for general robustness in *D*. *melanogaster*.

## Materials and Methods

### The *D. melanogaster* population

For the experiments, we used a mass bred *D*. *melanogaster* population. This population was set up in 2010 using the offspring of 589 inseminated females caught at Karensminde fruit farm in Odder, Denmark (55°56042.46N, 10°12045.31E). The population was maintained at a size >1000 individuals on standard *Drosophila* agar-sugar-yeast-oatmeal medium (for recipe see^32^) at 25 ± 1 °C and 12-h light/12-h dark cycles for approximately 150 generations prior to performing the experiments reported here.

### Impact of age on starvation, heat, and cold tolerance

Flies being 3–5 days old were allowed to lay eggs on teaspoons with standard food. From these spoons, 40 eggs were collected into each of 30 vials (27 mL) with 7 mL standard food. Flies emerging from these vials were collected as virgins and separated into males and females when maximum 8 hours old. Male and female flies from the 30 vials were pooled within sex and then transferred to vials with 3 mL medium with 20 male or female flies in each of 25 vials. This procedure secured that a random subset of flies from the 30 vials they developed in were transferred to each of the 25 vials per sex. Every 3rd day, flies were tipped into new vials with 3 mL fresh standard medium. At age 3, 19 and 35 days 20 male and 20 female flies were tested for starvation, heat (CTmax) and cold (CTmin) resistance, respectively. Flies used for tests from all age-classes constituted a random group of flies from the 25 vials per sex.

Starvation resistance was tested by placing flies individually in 27 mL vials (closed with foam stoppers) containing 4 mL of a water and agar (1.5%) solution. Every 8 hours the number of dead flies was counted until all flies had died. Heat and cold resistance were tested in so-called ramping assays where temperatures are gradually increased or decreased from a starting temperature of 25 °C^33^. The temperatures at which no further movement of flies were observed constituted their Critical Thermal maximum or minimum (CTmax and CTmin, respectively). Flies were placed individually into sealed 6 mL glass vials and submerged into a glass tank containing a 25 °C liquid (water for CTmax and a mixture of ethylene glycol and water (1:1 vol/vol) for CTmin). The temperature of the water was increased with a rate of 0.1 °C/min for assessment of CTmax. When CTmin was assessed, the temperature of the liquid in the glass tank was decreased from 25 °C with a rate of 0.1 °C/min. The flies were continuously monitored in intervals of 2–3 min and the temperature where no movement could be induced with a flashlight and a gentle knocking on the vials with a stick, was noted as the upper or lower thermal limit (CTmax and CTmin). We interpret a high CTmax and a low CTmin as indicating high heat and cold tolerance, respectively.

### Samples for mtDNA CN – effect of age

Similar to the procedure used to generate flies for the stress resistance assays, 3–5 days old flies were allowed to lay eggs on teaspoons with standard food. Forty eggs were collected from these spoons into each of 45 vials (27 mL) with 7 mL standard medium. When flies emerged from these vials they were collected across all 45 vials as virgins when max 8 hours old and separated into males and females. Random samples of males and females were distributed into 30 vials with 20 individuals in each. Every 3rd day, flies were tipped onto new vials with 3 mL fresh standard medium. Male and female flies were collected across all vials (with an approximate equal contribution from each vial) and snap frozen in liquid nitrogen and stored at −80 °C when 3, 19 and 35 days old. Three replicates of 50 flies were frozen for mtDNA CN assessment from each combination of sex and age (3 replicates of 50 flies per sex and age yielding a total of 18 samples).

### Mitochondrial DNA CN measurements in *D. melanogaster*

DNA was extracted from fly samples at Eurofins GenoSkan A/S, Tjele, Denmark using DNeasy® Blood & Tissue Kit (Qiagen), following a protocol for insect tissues according to manufacturer’s instructions. Mitochondrial DNA CN analyses were performed in house using triplicate samples and SYBR® Green technology (Applied Biosystems)^23^. One 100 bp PCR was targeted to the mitochondrial NADH dehydrogenase 4 L gene (ND4L) using the primer sequences 5′-TAAGAAAATTCCGAGGGATTCA-3′ for the forward primer and 5′-GGTCGAGCTCCAATTCAAGTTA-3′ for the reverse primer. Primers were originally described in Mutlu^34^. To quantify the amount of mtDNA another 126 bp PCR targeted to the nuclear rosy gene was used. The primer sequence for the forward primer was 5′-GGTGGTGAGCCTGTTCTTCAAG-3′ and for the reverse primer it was 5′-ACTGGTGTGTGGAATGTCTCGG-3. These primers were originally described in Wu *et al*.^35^. The reactions were performed in a high throughput 96-plate format using a StepOne instrument (Applied Biosystems) and a total volume of 10 µL including 1x Fast SYBR® Green Master Mix (Applied Biosystems), 5 µM of each of the primers and 2 ng of DNA. The amplification was preheated at 95 °C for 20 sec. followed by a 40 cycle program of 0.3 sec. at 95 °C, 15 sec. at 52 °C and 30 sec. at 72 °C. Each DNA sample was assayed in triplicates using either the ND4L primers or rosy primers in parallel reactions. For each 96-plate, a DNA control sample from the same individual and a ‘no template control’ was added in triplicates. The mtDNA copy number was calculated by the formula 2^(ctrosy median–ctND4L median)^ with ct being a threshold cycle that reflects the intersection between an amplification curve and a threshold line. The median of the three replicated values was calculated and these values were used in subsequent analyses of data.

### Hypotheses and statistical analyses

The following two hypotheses were tested: (1) The ability to cope with heat (CTmax), cold (CTmin) and food shortage (starvation) decreases with age and more so in males than in females, and (2) mtDNA CN is decreased at old age and more so in male compared to female flies. Data on all traits (CTmax, CTmin, starvation and mtDNA CN) were log-transformed in order to improve normalization of distributions, to homogenize the variance, and to linearize non-linear relationships. Both hypotheses were investigated by testing the main effects of age and sex and their interaction effects on the above traits in linear regression models. With sex coded as 0 for males and 1 for females, the main effect of age captures the effect of age in males and the interaction term captures the effect of age in females. All statistical analyses were performed using the software PAST v.2.12^36^.

## Results

### CTmin, CTmax, and starvation resistance in male and female flies of different age

**Figure 1:**
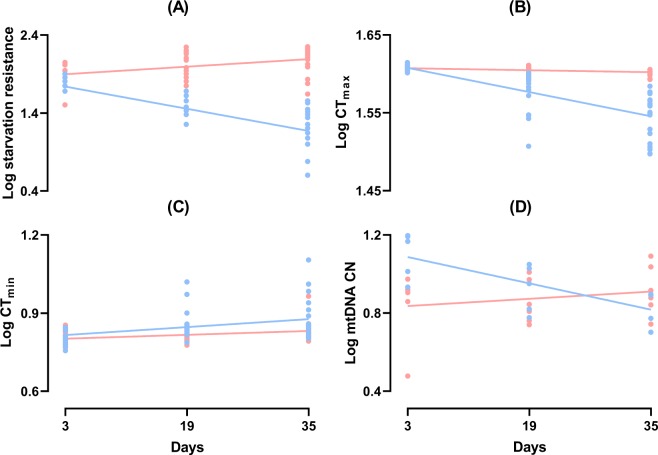
(**A**) Least square linear regression of log starvation resistance in hours (Log Starvation resistance) in male (blue) and female (red) *D*. *melanogaster* with the age of 3, 19 and 35 days. (**B**) Least square linear regression of log heat resistance (temperature) (Log CTmax) in male (blue) and female (red) *D*. *melanogaster* with the age of 3, 19 and 35 days. (**C**) Least square linear regression of log cold resistance (temperature) (Log CTmin) in male (blue) and female (red) *D*. *melanogaster* with the age of 3, 19 and 35 days. (**D**) Least square linear regression of the log-transformed mitochondrial DNA copy number (Log mtDNA CN) in male (blue) and female (red) *D*. *melanogaster* with the age of 3, 19 and 35 days.

The ability to tolerate periods without food (starvation) is reduced markedly with age in male flies as shown by the negative slope with age (p = 9.15e-19) (Table 1, Fig. 1A), whereas increasing age had a positive influence on starvation tolerance in females as indicated by the interaction effect between age and sex (p = 1.89e-17) (Table 1, Fig. 1A). Sex alone did not have a significant effect on starvation tolerance (p = 0.12).

**Table 1:** Linear regression results for the effects of age, sex and their interaction on CTmax, CTmin, starvation tolerance, and mtDNA CN.

Traits and Variables	Estimate	Std. Error	t value	Pr(>|t|)
**CTmax**				
Age	−1.938e-03	1.565e-04	−12.380	7.914e-23
Sex	−5.915e-03	5.165e-03	−1.145	2.546e-01
Age:Sex	1.780e-03	2.228e-04	7.990	1.203e-12
**CTmin**				
Age	1.907e-03	4.497e-04	4.242	4.511e-05
Sex	−1.111e-02	1.468e-02	−0.757	4.511e-01
Age:Sex	−9.777e-04	6.401e-04	−1.528	1.294e-01
**Starvation**				
Age	−1.775e-02	1.675e-03	−10.598	9.154e-19
Sex	8.599e-02	5.462e-02	1.574	1.182e-01
Age:Sex	2.379e-02	2.369e-03	10.040	1.894e-17
**mtDNA CN**				
Age	−8.421e-03	2.516e-03	−3.347	2.272e-03
Sex	−2.835e-01	7.855e-02	−3.609	1.144e-03
Age:Sex	1.073e-02	3.407e-03	3.151	3.760e-04

For heat resistance (CTmax), males became less tolerant with increasing age as we observed a significant negative slope with age in the regression (p = 7.91e-23), whereas for females, their resistance to heat is not changed with age suggested by the highly significant positive interaction effect (p = 1.20e-12) (Table 1, Fig. 1B). Likewise, sex itself does not seem to affect heat resistance (p = 0.25).

Both males and females had reduced cold tolerance (higher CTmin) at older age. This is evident from the significant positive slopes for the main effect of sex (Table 1, Fig. 1C). Males and females became equally more cold susceptible (less cold tolerant) with increasing age (Table 1; Fig. 1C).

### mtDNA CN in male and female flies of different age

The same model as for stress resistance traits was applied to the mtDNA data to test hypothesis 2. In general, we observed a significantly lower level of mtDNA CN in females compared to males, as shown by the negative coefficient of sex (p = 0.001). mtDNA CN decreases significantly with increasing age in males (p = 0.002) but the pattern is reversed in females as suggested by the significant positive interaction term (p = 0.004) (Table 1 and Fig. 1D).

## Discussion

Sex specific aging and lifespan is a common phenomenon across many species including *D*. *melanogaster* where lifespan expectancy is higher for the female sex^10,11,37^. The reasons for sexual dimorphism in lifespan remain poorly understood although several hypotheses including differential vulnerability to environmental stress, differential intensity of sexual selection, distinct patterns of parental care, and sex specific genetic architectures of the investigated traits, have been proposed^11,23^. Here we investigate whether male and female *D*. *melanogaster* have differential stress resistance and whether mtDNA CN varies with age and sex. In summary, we provide evidence that stress resistance changes at old age in a sex and trait specific manner. Our data suggest that males are typically less stress resistant compared to females and that reductions in stress resistance with increasing age are more pronounced in males (Fig. 1A–C). In addition, we observe that mtDNA CN decreases markedly with age in males, whereas this is not the case in females (Fig. 1D). Thus in males we observe a clear association between a reduced ability to cope with environmental stress with increasing age and decreases in mtDNA CN. This is in contrast to females where mtDNA CN is not reduced at old age suggesting that within the age classes investigated mtDNA is a poor indicator of age dependent stress tolerance in females.

Our finding that females are generally more stress resistant than males is a common observation in studies on *Drosophila* spp.^15,38,39^ and longer lifespan of females has also been observed previously^15,37^. In an attempt to understand the genetic architecture of these observations candidate genes explaining phenotypic variation in lifespan and stress resistance within and between populations of *D*. *melanogaster* and other model organisms have been identified^40–43^. Studies have also pinpointed genes that have an impact on both lifespan and stress resistance including the stress traits assessed in this study (thermal extremes and starvation)^43,44^. For example Shaposhnikov *et al*.^44^ demonstrated that overexpression of DNA repair genes affected both lifespan and stress resistance in *D*. *melanogaster* suggesting a shared genetic basis. These genes included genes involved in recognition of DNA damage (homologs of HUS1, CHK2), nucleotide and base excision repair (homologs of XPF, XPC and AP-endonuclease-1), and repair of double-stranded DNA breaks (homologs of BRCA2, XRCC3, KU80 and WRNexo). Also Sørensen *et al*.^41^ suggested shared genetic mechanisms between longevity and stress resistance. By investigating gene expression in replicate *D*. *melanogaster* lines selected for increased longevity, desiccation and starvation resistance, respectively, they showed high similarities in gene expression patterns across these selection regimes emphasizing a shared genetic architecture. A connection between resistance to starvation and desiccation and increased longevity has also been found at the functional phenotypic level^45,46^. Thus, we have knowledge about the shared genetic architectures of stress resistance and lifespan but variation in these genes may only explain sexual dimorphism if they are sex linked or determining phenotypic differences between sexes through other mechanisms such as by sex limited or sex influenced expression of genes. Fewer studies have investigated this aspect but those that have often find highly sex specific genetic architectures of stress resistance and longevity in *D*. *melanogaster* and these differences are not limited to sex linked genes, suggesting a complex interplay between sex, environmental stress resistance and longevity^12,26,47^.

We find that the impact of aging on environmental stress tolerance is highly sex specific. Starvation resistance does not decrease with age in females; on the contrary, old females are better at coping with food shortage compared to young females. In males, the ability to tolerate periods without food is reduced markedly at old age. The same pattern was found when looking at the capacity of males and females to tolerate heat at old age, as old males showed a reduced capacity to tolerate heat, while for females the impact of age was minor. The trend that males were more vulnerable with increasing age was also observed for resistance to cold temperatures (although not significant). Here both sexes had decreased cold resistance at old age (increased CTmin). In contrast to our measures of heat and starvation resistance, where we obtain information about respectively temperature and time at which death occurs, CTmin is a measure of the temperature at which flies enter into chill coma. If brought back to benign temperatures flies will typically recover. However, across *Drosophila* species we know that measures of cold tolerance are highly correlated and a strong association between CTmin and assays employing lethality as the endpoint exists^48^. Thus interpreting observations from the three stress tolerance assays in a similar way is in our opinion justified.

It is well established that females live longer than males in *D*. *melanogaster*^11^. Unpublished work (T.N. Kristensen and V. Loeschcke) on the population of *D*. *melanogaster* investigated here confirm this. These data show that while no mortality is observed in neither of the sexes at 5 days of age, the proportion of females and males surviving to day 19 and 35 differ markedly. At 19 days of age, 2% and 7% of females and males, respectively, have died, whereas these numbers are 7% and 40% at 35 days. Thus, e.g. 35 days old females seem to be physiologically younger than males at the same chronological age.

Based on published literature and observations from the present study we can speculate on the reasons for the trait dependent sexual dimorphism observed. We know from other studies that activity is reduced in old *D*. *melanogaster*^49,50^ and that males are more active across age classes compared to female *D*. *melanogaster*^51,52^. There is also evidence that thermal stress has a significant negative impact on organismal energy reserves in *D*. *melanogaster*^53^, and that exposure to acute high temperatures increases both locomotor activity and metabolic rate, whereas starvation and cold exposure lead to reductions in both^54–56^. Combined with the notable observation from the current study that mtDNA CN is reduced with age in males but not in females this suggests that differences in metabolic demand and efficiency in male and female flies is key to understand sexual dimorphism in lifespan and trait specific and sex dependent responses to aging. Interpreting results on mtDNA CN from males could potentially be difficult if sperm carried mitochondria because age dependent mtDNA CN with increasing age could then reflect decreasing sperm production at old age. However, sperm from mature *D*. *melanogaster* males does not have mitochondria and therefore we do not consider this a problem^57^.

The comparisons between mtDNA CN and stress resistance in male flies indicated that there is a clear association between the drop of the mtDNA CN median value with age and the drop in heat, cold and starvation resistance. Based on this result we propose that mtDNA CN variation is important for explaining sexual dimorphism in lifespan and trait- and sex specific consequences of aging and that mtDNA CN may be a suitable biomarker for physiological status and general health across species. The constancy (minor increase with age) of mtDNA CN across age-classes in females and the age-dependent decline of mtDNA CN in males suggest that female *D*. *melanogaster* are better than males able to control and maintain mitochondrial function at old age. Any causation between sex specific impact of aging on stress resistance and mtDNA CN is not provided in this study but this result does support that mitochondrial functioning is key to understand sex specific lifespan and robustness at old age. In providing further knowledge on this we advocate, that studies should include older females, e.g. by testing females at an age where their physiological age is similar to a 35 day old male. According to our pilot data (T.N. Kristensen and V. Loeschcke) obtained on the same population as used in this study this could e.g. be done by including females having an age where approximately 60% of the cohort remains alive as is the case for males being 35 days old in the current study. This would provide further knowledge on age related declines in stress resistance in females and whether mtDNA CN is also reduced in old females but at an older chronological age.

The observed changes of mtDNA CN with age and sex coupled with the results from the stress resistance assays lead us to conclude that high sexual dimorphism is observed for ecologically important stress traits and for a measure of metabolism and energetic efficiency, namely mtDNA CN. These results suggest that sex specific abilities to cope with aging might be more important for our understanding of sexual selection and adaptation than hitherto assumed. Based on our results we also conclude that mtDNA CN might be a biomarker for physiological age and suggest that more studies should investigate the importance of mtDNA CN for understanding sexual dimorphism and age dependent abilities to cope with extreme environmental conditions.
